# Anti-inflammatory, wound healing and antioxidant potential of compounds from *Dioscorea bulbifera* L. bulbils

**DOI:** 10.1371/journal.pone.0243632

**Published:** 2020-12-11

**Authors:** Prapaporn Chaniad, Supinya Tewtrakul, Teeratad Sudsai, Supat Langyanai, Kantarakorn Kaewdana

**Affiliations:** 1 School of Medicine, Walailak University, Nakhon Si Thammarat, Thailand; 2 Faculty of Pharmaceutical Sciences, Prince of Songkla University, Hat-Yai, Songkhla, Thailand; North-Eastern Hill University, India, INDIA

## Abstract

**Background:**

*Dioscorea bulbifera* L. (Dioscoreaceae) has been traditionally used in Thai folk medicine as a diuretic and anthelmintic, for longevity preparations, and for wound and inflammation treatment. This plant is also commonly used in traditional Indian and Chinese medicines in the treatment of sore throat, gastric cancer, rectal carcinoma and goiters. However, the wound healing effects of the active compounds in this plant have not been investigated.

**Objective:**

This study aimed to identify compounds responsible for the wound healing activity of *D*. *bulbifera* and determine their potential anti-inflammatory and antioxidant activities.

**Methods:**

Crude extracts of *D*. *bulbifera* bulbils, their derived fractions and eleven purified compounds were tested for anti-inflammatory activity against LPS-induced NO production in RAW264.7 macrophages. The wound healing effects were evaluated via cell proliferation and migration assays using human dermal fibroblasts (HDFs), and the antioxidant effects were determined using 2,2-diphenyl-1-picrylhydrazyl (DPPH) and hydroxyl radical (^•^OH) scavenging activity assays.

**Results:**

15,16-Epoxy-6α-*O*-acetyl-8β-hydroxy-19-nor-clero-13(16),14-diene-17,12;18,2-diolide (**2**), (+)-catechin (**5**), quercetin (**6**) and myricetin (**11**) exhibited significantly potent wound healing effects and promoted marked cell proliferation, resulting in % viabilities of 107.4–137.6, 121.1–151.9, 98.0–131.9, 90.9–115.9, respectively. Among them, (+)-catechin produced the highest % cell migration, resulting in 100.0% wound closure sooner (at day 2) than the other compounds. In addition, 1 μg/ml (+)-catechin significantly increased fibroblast migration by 2.4-fold compared to that in the control after 24 h. Regarding anti-inflammatory properties, kaempferol (**7**) and quercetin (**6**) decreased (*p* < 0.005) NO production, with IC_50_ values of 46.6 and 56.2 μM, respectively. In addition, the crude extracts, solvent fractions and flavonoid compounds were also found to possess marked antioxidant activity in both DPPH and ^•^OH radical scavenging assays.

**Conclusions:**

These findings provide more evidence to support the traditional use of *D*. *bulbifera* for the treatment of wounds and inflammation.

## Introduction

Inflammation is the immune system’s response to infectious agents, toxic compounds, or injury and acts by removing injurious stimuli and initiating the healing process. During acute inflammatory responses, cellular and molecular events and interactions efficiently minimize impending injury or infection. This mitigation is a coordinated and active process to restore tissue integrity and function [1]. Dysregulation of the inflammatory response may lead to prolonged inflammation, possibly resulting in host tissue damage and pathological chronic inflammation [2].

Wound healing is a complex and dynamic physiological process that results in the recovery of structural and functional tissue integrity [3]. Many cell types and mediators are involved in the normal process of wound healing to restore barrier function and prevent further damage or infection [4]. Unhealable wounds have a significant impact on the health and quality of life of patients and cause pain, function loss, mobility loss, depression, and anxiety, prolong hospitalization and increase morbidity and mortality [5].

The wound healing process can be characterized by three overlapping inflammatory, proliferative and remodeling phases that repair and organize structures to increase the tensile strength of the damaged tissue partially or completely depending on the severity of the wound [6]. It is well known that when wounding occurs, the short-term process of inflammation caused by the release of inflammatory mediators and radical oxygen species via macrophages mainly impairs and delays the process of wound repair. Thus, the inhibition of reactive radical production is an important consideration in the recruitment of fibroblasts, which are attracted to the site to initiate the proliferative phase of repair or the wound healing process [7].

*Dioscorea bulbifera* L. (family, Dioscoreaceae) is commonly known as air potato. This plant is widely distributed in many parts of Thailand and locally known in Thailand as ‘Wan Phra Chim’. Its bulbils have been traditionally used in Thai folk medicine as a diuretic and anthelmintic, in longevity preparations, and for wound and inflammation treatment [8]. In traditional Indian and Chinese medicine, this plant is commonly also used to treat sore throat, gastric cancer, rectal carcinoma and goiters [9]. Moreover, in Cameroon and Madagascar, pounded bulbs are applied to abscesses, boils, and wound infections [10].

Crude extracts of this plant have been found to possess antihyperglycemic, antidyslipidemic, antimicrobial, antidiabetic, analgesic and anti-HIV-1 integrase activities [9–13]. Moreover, *D*. *bulbifera* extract has reported to present a potential anti-inflammatory effect that reduces paw edema [10] and produce a high rate of wound contraction [14]. Some compounds isolated from this plant, such as quercetin and kaempferol, have also been found in various medicinal plants that have previously been reported to possess wound healing effects [15,16]. However, limited information is available on the wound healing effect of *D*. *bulbifera*. Therefore, this study was conducted to identify compounds responsible for the wound healing activity of *D*. *bulbifera* and to determine their potential anti-inflammatory and antioxidant activities.

## Materials and methods

### Chemicals

Roswell Park Memorial Institute 1640 medium (RPMI-1640), Dulbecco's modified Eagle medium (DMEM), and 3-(4,5-dimethylthiazol-2-yl)-2,5-diphenyltetrazolium bromide (MTT) were purchased from Gibco (Life Technologies, Paisley, Scotland). L-Nitroarginine (L-NA), caffeic acid phenethyl ester (CAPE), indomethacin, lipopolysaccharide (LPS), fetal bovine serum (FBS), aspirin, ibuprofen, phosphate-buffered saline (PBS), 2,2-diphenyl-1-picrylhydrazyl (DPPH), ethylenediaminetetraacetic acid (EDTA), and L-ascorbic acid were purchased from Sigma-Aldrich (USA). All other chemicals were purchased from Merck (Darmstadt, Germany).

### Plant material and isolation

Bulbils of *D*. *bulbifera* were collected from Uttaradit Province, Thailand, in 2011. A voucher specimen (SKP 062040201) was identified by a botanist of the Forest Herbarium, Department of National Parks, Wildlife and Plant Conservation, Thailand, and has been deposited in the Department of Pharmacognosy and Pharmaceutical Botany, Faculty of Pharmaceutical Sciences, Prince of Songkla University, Hat-Yai, Songkhla, Thailand.

**Fig 1:**
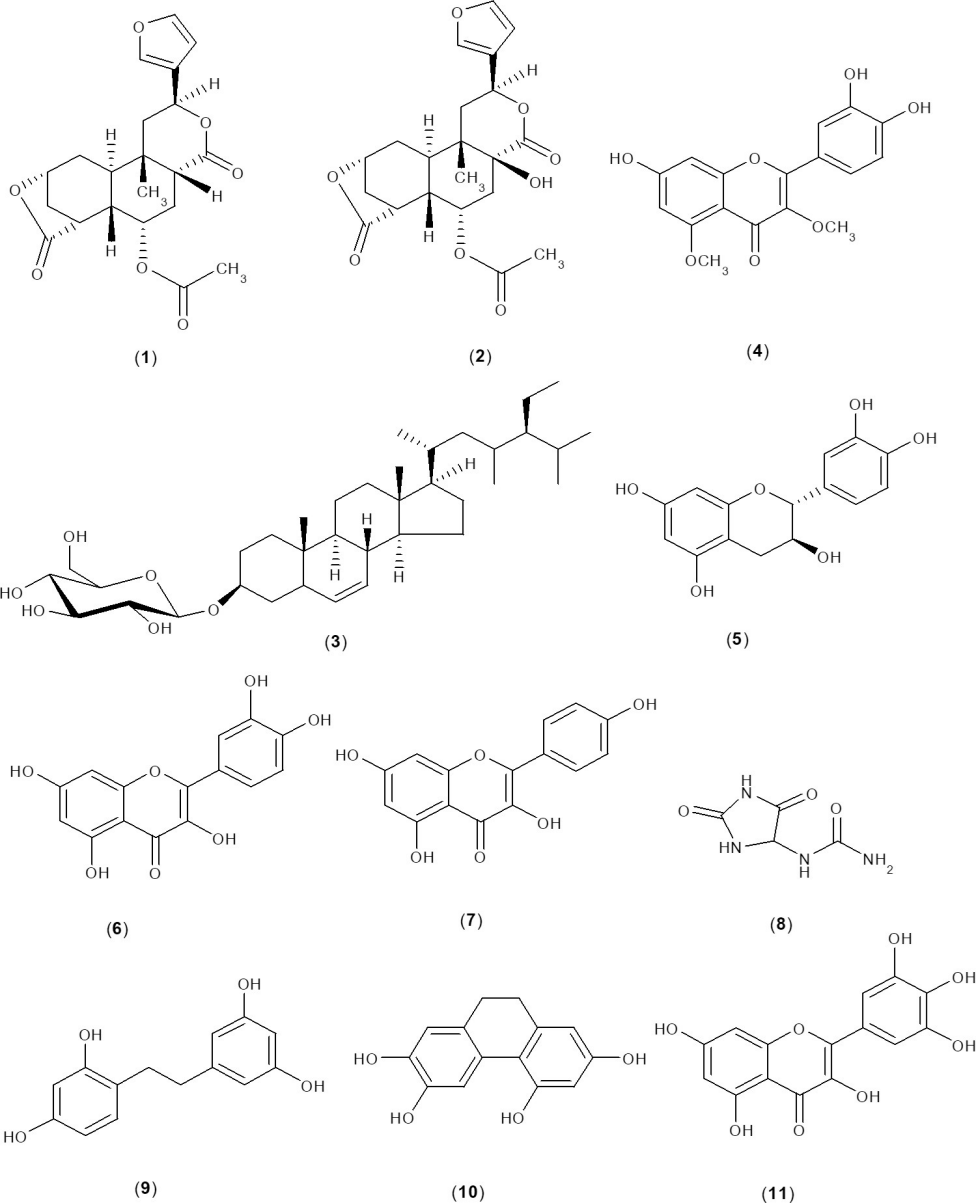
Structures of the compounds isolated from bulbils of *D*. *bulbifera*.

According to previous reports by our research group, *D*. *bulbifera* bulbils were extracted, the extracts were fractionated, and compounds were isolated. Briefly, the powder of bulbils was separately extracted with ethanol by maceration at room temperature and extracted with water by the reflux method to produce crude ethanol and water extracts. Ethanol extract was subsequently partitioned with hexane, chloroform, ethyl acetate and water to obtain chloroform, ethyl acetate and water fractions without residue from the hexane fraction. These fractions were further separated by chromatography methods to give purified compounds [13,17]. In the current study, the biological activities of the crude extracts (EtOH and water extracts), their derived fractions (CHCl_3_, EtOAc and water fractions) and eleven compounds (**1**–**11**) were investigated. The isolated compounds consisted of 8-epidiosbulbin E acetate (**1**), 15,16-epoxy-6α-*O*-acetyl-8β-hydroxy-19-nor-clero-13 (16), 14-diene-17,12;18,2-diolide (**2**), sitosterol-β-D-glucoside (**3**), 3,5-dimethoxyquercetin (**4**), (+)-catechin (**5**), quercetin (**6**), kaempferol (**7**), allantoin (**8**), 2,4,3',5'-tetrahydroxybibenzyl (**9**), 2,4,6,7-tetrahydroxy-9,10 dihydrophenanthrene (**10**) and myricetin (**11**) (Fig 1).

### Anti-inflammatory activity

#### Anti-NO production assay

To evaluate the anti-inflammatory activity, the inhibition of nitric oxide (NO) production was evaluated according to a previous report [18]. Briefly, RAW264.7 cells were seeded into sterile 96-well plates (1x10^5^ cells/well) and incubated for 1 h at 37°C in a humidified incubator containing 5% CO_2_. The cells were then cultured in RPMI-1640 medium containing lipopolysaccharide (LPS, 100 ng/ml) together with the test sample at various concentrations (3–100 μM). After 24 h of incubation, the nitrite (NO_2_^–^) concentration in the culture medium was determined as an indicator of NO production using Griess reagent to assay the accumulation of NO_2_^–^, a stable metabolite of NO. The absorbance was measured using a microplate reader at 570 nm. In this study, a NO synthase inhibitor (L-nitroarginine, L-NA), an inhibitor of the nuclear translocation of NF-κB (CAPE) and a nonsteroidal anti-inflammatory drug (NSAID; indomethacin and aspirin) were used as positive controls.

#### Cell viability test

The cytotoxicity of the test compounds after 24 h of incubation was determined according to a previously reported method [18]. A volume of 10 μl of MTT solution (5 mg/ml in PBS) was added to each well of 96-well plates and further incubated in a CO_2_ incubator for 4 h. The formazan products generated by MTT reduction were dissolved in dimethyl sulfoxide (DMSO). Finally, the medium was removed, and 100 μl of DMSO was added to each well and thoroughly mixed by gentle tapping on the test plate. The absorbance of the formazan solution was measured at a wavelength of 570 nm using a microplate reader. The test compounds were considered cytotoxic when the viability of the compound-treated group was less than 80% of that in the control (1% DMSO-treated) group.

### Wound healing assay

#### Cell proliferation and viability

The cell proliferation and viability of human dermal fibroblasts (HDFs) seeded at 1x10^4^ cells/well into 96-well plates in DMEM containing 10% FBS were evaluated. After 24 h, the cells were exposed to different concentrations (1–100 μM) of test samples and then incubated for 48 h at 37°C in a humidified atmosphere containing 5% CO_2_. MTT solution (10 μl, 5 mg/ml) was added directly to the medium in each well, and the plate was then incubated at 37°C for 4 h. All medium was then aspirated and replaced with DMSO, and the optical density at 570 nm was recorded. The percentage of cell proliferation was calculated and compared to that of the negative control [18].

%Cellviability=(Absorbanceofsample/Absorbanceofcontrol)×100

#### Migration of HDFs

The migration of HDFs was examined using a wound healing method. Briefly, HDFs (5 x 10^4^ cells/ml) in DMEM containing 10% FBS were seeded into each well of 24-well plates and incubated at 37°C with 5% CO_2_. After the confluent monolayer of HDFs had formed, a sterile pipette tip was used to generate two horizontal scratches (left and right) in each well. Any cellular debris was removed by washing with PBS and replacement with 1 ml of fresh medium in the absence or presence of the test sample. Photographs were taken on day 0, the plates were then incubated at 37°C with 5% CO_2_, and photographs were taken on each day from day 1 to day 3. To determine the migration of HDFs, the images were analyzed using ImageJ. The percentage of the closed area was measured and compared with the value obtained before treatment (day 0). An increase in the percentage of closed area indicates cell migration [18].

### Antioxidant activity

#### DPPH radical scavenging assay

The antioxidant activity of the samples against DPPH radicals was evaluated according to the method previously described [19] with slight modifications. A total of 75 μl of sample solution at various concentrations (1–100 μM) was mixed with 125 μl of DPPH (0.1 mM in ethanol) in a 96-well plate. The reaction mixture was incubated in the dark at room temperature for 30 min. Then, the absorbance at 517 nm of the mixture was determined using a microplate reader. A control solution was prepared by mixing absolute ethanol and DPPH solution. The antioxidant efficacy of all samples was compared with that of ascorbic acid, a positive control. The experiment was carried out in triplicate independently.

#### Hydroxyl radical (^•^OH) scavenging activity

This assay was carried out according to a previous method [20] with slight modifications. Briefly, the reaction mixtures contained the following reagents in a final volume of 1.2 ml: FeCl_3_ (100 μM), EDTA (104 μM), hydrogen peroxide (H_2_O_2_) (1.0 mM), 2-deoxy-D-ribose (2.8 mM) and various concentrations of test samples (1–100 μg/ml for extracts and its fractions; 1–100 μM for pure compounds). After incubation at 37°C for 1 h, 500 μl of 10% TCA and 500 μl of 5% TBA were added and incubated at 95°C for 30 min to develop the color. After cooling at room temperature for 10 min, the absorbance was measured at 532 nm. Butylated hydroxytoluene (BHT) was used as a positive control.

### Statistical analysis

For the statistical analyses, all data were expressed as the mean ± S.E.M. of three determinations. The 50% inhibitory concentration (IC_50_) values were calculated using Microsoft Excel. Data analysis was performed by one-way analysis of variance (ANOVA), followed by Duncan’s test. In the study, *p* values < 0.05 were considered significant.

## Results and discussion

### Anti-inflammatory activity

The inflammatory phase is the first and essential stage in the wound healing process [21]. In this process, NO is an important biomolecule that causes vasodilation and cellular migration [22]. Therefore, suppression of NO may be a good therapeutic target to promote wound healing. Accordingly, in the present study, the anti-inflammatory activity of crude extracts, their fractions and isolated compounds from *D*. *bulbifera* bulbils was assessed in RAW264.7 macrophage cells induced by LPS, a major biological endotoxin able to increase NO production. The results revealed that the extracts and its fractions of *D*. *bulbifera* possessed mild anti-inflammatory activity. The isolated compounds (**1**–**11**) were also evaluated for their anti-NO activity, and the results are shown in Table 1. Among them, myricetin (**11**) exhibited the highest inhibitory activity, with an IC_50_ value of 39.0 μM, followed by kaempferol (**7**) and quercetin (**6**), with IC_50_ values of 46.6 and 56.2 μM, respectively. However, 2,4,3',5'-tetrahydroxybibenzyl (IC_50_ = 96.3 μM) and other compounds (IC_50_ > 100 μM) exhibited weak activity. A cytotoxic effect was observed for only sitosterol-β-D-glucoside (**3**) at concentrations of 10, 30 and 100 μM. Remarkably, the inhibitory effect of the active compounds (**11** and **7**) was higher than that of a positive control, ibuprofen (IC_50_ = 54.5 μM), and comparable to that of aspirin (IC_50_ = 43.2 μM) and indomethacin (IC_50_ = 46.5 μM), which are clinically used NSAIDs.

**Table 1:** Anti-nitric oxide production in RAW264.7 cells exposed to extracts, their derived fractions and isolated compounds from *D*. *bulbifera*.

No	Sample	% Inhibition at various concentrations (μM)				IC_50_ (μM)
		0	10	30	100	
1^c^	EtOH extract	0.0 ± 1.8	15.7 ± 0.7	26.2 ± 4.3*	52.5 ± 0.4*	94.1 ± 0.8*
2^c^	Water extract	0.0 ± 1.8	1.4 ± 1.8*	7.1 ± 0.6*	22.2 ± 0.5*	> 100
3^c^	CHCl_3_ fraction	0.0 ± 1.8	3.6 ± 1.1	6.4 ± 0.8*	26.6 ± 1.7*	> 100
4^c^	EtOAc fraction	0.0 ± 1.8	10.4 ± 2.4	14.4 ± 1.2*	37.5 ± 0.9*	> 100
5^c^	Water fraction	0.0 ± 1.8	-0.9 ± 0.5*	-0.6 ± 0.6*	7.0 ± 1.3*	> 100
6	8-Epidiosbulbin E acetate (**1**)	0.0 ± 1.9	-6.3 ± 1.2*	3.4 ± 1.0*	26.3 ± 1.9*	> 100
7	15,16-Epoxy-6α-*O*-acetyl-8β-hydroxy-19-nor-clero-13(16),14-diene-17,12;18,2-diolide (**2**)	0.0 ± 1.9	-3.0 ± 1.6*	-0.1 ± 2.6*	20.4 ± 1.6*	> 100
8	Sitosterol-β-D-glucoside (**3**)	0.0 ± 1.9	-0.8 ± 4.0*^b^	3.1 ± 2.8*^b^	17.6 ± 4.5*^b^	> 100
9	3,5-Dimethoxyquercetin (**4**)	0.0 ± 1.9	5.5 ± 1.5 *	17.6 ± 5.1*	31.6 ± 0.9*	> 100
10	(+)-Catechin (**5**)	0.0 ± 1.9	0.2 ± 0.9*	4.3 ± 1.7*	19.5 ± 2.8*	> 100
11	Quercetin (**6**)	0.0 ± 1.9	6.5 ± 1.5 *	28.8 ± 0.8	67.5 ± 1.5*	56.2 ± 0.8*
12	Kaempferol (**7**)	0.0 ± 1.9	6.3 ± 1.6 *	25.7 ± 1.5*	79.3 ± 2.4	46.6 ± 1.5
13	Allantoin (**8**)	0.0 ± 1.8	5.6 ± 4.0 *	9.1 ± 0.7*	33.2 ± 2.0*	> 100
14	2,4,3',5'-Tetrahydroxybibenzyl (**9**)	0.0 ± 1.8	4.6 ± 1.5 *	19.5 ± 1.1*	51.0 ± 0.7*	96.3 ± 1.1*
15	2,4,6,7-Tetrahydroxy-9,10-dihydrophenanthrene (**10**)	0.0 ± 1.8	6.5 ± 1.4 *	15.4 ± 0.5*	42.5 ± 3.0*	> 100
16	Myricetin (**11**)	0.0 ± 1.8	12.1 ± 1.0	35.2 ± 1.4	82.3 ± 1.1*	39.0 ± 1.4
17	Aspirin	0.0 ± 2.0	7.0 ± 3.8*	29.4 ± 1.9	81.8 ± 0.6*	43.2 ± 1.5
18	Ibuprofen	0.0 ± 2.0	6.1 ± 2.0*	27.7 ± 2.5	75.2 ± 1.7	54.5 ± 2.0*
19	Indomethacin	0.0 ± 2.0	12.3 ± 1.5	31.3 ± 1.5	74.0 ± 1.0	46.5 ± 1.2
20	CAPE	0.0 ± 2.0	54.4 ± 0.8*	88.6 ± 1.1*	95.5 ± 0.7*	9.3 ± 0.4*
21	L-NA	0.0 ± 2.0	12.0 ± 1.8	29.0 ± 2.1	89.4 ± 0.8*	37.7 ± 1.8*

*Statistically significant difference compared to indomethacin, *p* < 0.05 (mean ± S.E.M. of three determinations).

^b^Cytotoxic effect was observed.

^c^Concentration of treated samples and IC_50_ unit expressed in μg/ml.

Regarding the other *Dioscorea* species, *D*. *alata* tuber extract has been reported to possess a potent anti-inflammatory effect by inhibition of the NO and TNF-α expression [23]. *D*. *batatas* peel extract decreased NO production and proinflammatory protein expression and decreased the level of ROS [24]. In addition, aerial bulblet extract of *D*. *japonica* was reported to exhibit anti-inflammatory activity, which may be attributed to its effect by the inhibition of nuclear factor kappa-B (NF-κB) and mitogen-activated protein kinase (MAPK) activation [25].

Regarding myricetin (**11**), our study is in accordance with a previous report that it inhibited the LPS-stimulated production of NO and the production of prostaglandin E_2_ (PGE_2_) and to decrease inducible nitric oxide synthase (iNOS) and cyclooxygenase-2 (COX-2) expression [26]. Regarding the NO inhibitory activity of active flavonoid compounds (**6**, **7** and **11**), the structure-activity relationship suggested that the C2-C3 double-bond and 4-oxo functional group of the C-ring are important factors responsible for the strong inhibition of COX-2 expression [27], and methoxylation at position 3, e.g., 3,5-dimethoxyquercetin, reduced the activity (**4**; IC_50_ > 100 μM) compared with that of quercetin (**6**; IC_50_ = 56.2 μM).

### Wound healing activity

#### Cell proliferation

As the cell proliferative phase progresses, fibroblasts become the predominant cells at the wound site that play an important role in wound contraction to restore the integrity of injured tissue [28]. Fibroblasts secrete the collagens and glycosaminoglycans for the new granulation tissue and subsequently affect the remodeling of the granulation tissue into mature dermis [29]. In the current study, HDFs, skin fibroblasts were used to investigate the ability of extracts, their derived fractions and purified compounds from *D*. *bulbifera* bulbils to promote cell proliferation.

Regarding the cell proliferation in the presence of extracts of *D*. *bulbifera* (1–100 μg/ml), the EtOH extract (E1) and water extract (E2) showed low cytotoxicity, with % cell viabilities in the range of 78.8–98.2 (E1) and 77.2–84.3 (E2) (Table 2), whereas the CHCl_3_ (F1), EtOAc (F2) and water (F3) fractions promoted cell proliferation, with % viabilities of 92.3–134.4 (F1), 102.9–129.7 (F2) and 103.3–144.4 (F3), respectively. *Aloe vera* gel, a positive control, resulted in a % cell viability (98.3–107.0) less than those of F1-F3 (Table 2).

**Table 2:** Percent HDF viability (cell proliferation) in the presence of extracts and fractions from *D*. *bulbifera* and *A*. *vera* gel.

Sample	% Viability of HDFs at various concentrations (mean ± S.E.M.) (μg/ml)					
	0	1	3	10	30	100
EtOH extract	100.00 ± 1.35	98.29 ± 2.33	96.12 ± 3.26*	94.01 ± 3.39*	91.87 ± 2.25*	78.83 ± 1.79*
Water extract	100.00 ± 1.35	84.34 ± 3.23*	84.23 ± 2.25*	77.24 ± 2.82*	83.70 ± 2.34*	79.71 ± 3.37*
CHCl_3_ fraction	100.00 ± 2.34	131.55 ± 2.66*	134.42 ± 1.47*	124.79 ± 2.21*	92.38 ± 2.86*	101.67 ± 2.31
EtOAc fraction	100.00 ± 2.34	119.17 ± 2.84*	124.80 ± 1.85*	129.71 ± 2.90*	125.15 ± 2.21*	102.94 ± 3.22*
Water fraction	100.00 ± 1.41	135.27 ± 3.27*	144.47 ± 3.25*	103.37 ± 3.36	133.44 ± 1.62*	132.80 ± 3.38*
*A*. *vera* gel	100.00 ± 0.76	98.38 ± 1.63	105.88 ± 4.72	107.00 ± 2.67	104.76 ± 4.21	107.03 ± 4.12

*Statistically significant difference between *A*. *vera* gel and the sample, *p* < 0.05 (mean ± S.E.M. of three determinations).

Cell proliferation in the presence of compounds isolated from *D*. *bulbifera* (1–100 μM) showed that **2**, **5**, **6** and **11** promoted marked cell proliferation, with % viabilities of 107.4–137.6, 121.1–151.9, 98.0–131.9, 90.9–115.9, respectively (Table 3). Therefore, these four compounds were tested in a HDF cell migration assay.

**Table 3:** Percent HDF viability (cell proliferation) of compounds from *D*. *bulbifera* and *A*. *vera* gel.

Sample	% Viability of HDFs at various concentrations (mean ± S.E.M.) (μM)					
	0	1	3	10	30	100
8-Epidiosbulbin E acetate (**1**)	100.00 ± 1.99	132.02 ± 3.87*	107.82 ± 4.71	108.96 ± 3.65	127.85 ± 4.31*	122.47 ± 4.28*
15,16-Epoxy-6α-*O*-acetyl-8β-hydroxy-19-nor-clero-13(16),14-diene-17,12;18,2-diolide (**2**)	100.00 ± 1.99	118.64 ± 2.87*	137.65 ± 4.51*	111.99 ± 4.76	113.04 ± 3.93	107.49 ± 3.14
Sitosterol-β-D-glucoside (**3**)	100.00 ± 1.19	123.93 ± 6.23*	97.76 ± 3.27	112.43 ± 2.96	103.90 ± 4.82	94.67 ± 4.01*
3,5-Dimethoxyquercetin (**4**)	100.00 ± 1.19	96.62 ± 1.34	104.13 ± 1.69	105.65 ± 2.59	110.98 ± 2.45*	108.33 ± 0.59
(+)-Catechin (**5**)	100.00 ± 1.52	143.20 ± 3.04*	151.90 ± 4.89*	130.86 ± 3.00*	137.47 ± 3.83*	121.15 ± 2.98*
Quercetin (**6**)	100.00 ± 1.41	98.03 ± 0.91	131.80 ± 2.76*	131.95 ± 1.56*	113.36 ± 2.49*	117.96 ± 3.33*
Kaempferol (**7**)	100.00 ± 2.44	92.71 ± 1.63	98.88 ± 2.43*	94.98 ± 1.94*	98.57 ± 1.13*	121.02 ± 3.10*
Allantoin (**8**)	100.00 ± 1.52	130.31 ± 1.85*	108.57 ± 4.19	106.10 ± 2.54	105.27 ± 3.98	111.89 ± 2.74
2,4,3',5'-Tetrahydroxybibenzyl (**9**)	100.00 ± 2.44	78.87 ± 3.98*	110.70 ± 1.09	111.32 ± 1.16	99.61 ± 1.34	105.65 ± 3.79
2,4,6,7-Tetrahydroxy-9,10 dihydrophenanthrene (**10**)	100.00 ± 2.08	109.01 ± 4.03*	108.96 ± 1.56	98.00 ± 3.22*	99.92 ± 3.83	111.82 ± 2.69
Myricetin (**11**)	100.00 ± 2.08	111.53 ± 1.05*	115.92 ± 1.16*	112.44 ± 4.33	110.34 ± 1.20	90.90 ± 1.47*
*A*. *vera* gel (μg/ml)	100.00 ± 0.76	98.38 ± 1.63	105.88 ± 4.72	107.00 ± 2.67	104.76 ± 4.21	107.03 ± 4.12

*Statistically significant difference between *A*. *vera* gel and the sample, *p* < 0.05 (mean ± S.E.M. of three determinations).

#### Cell migration

**Fig 2:**
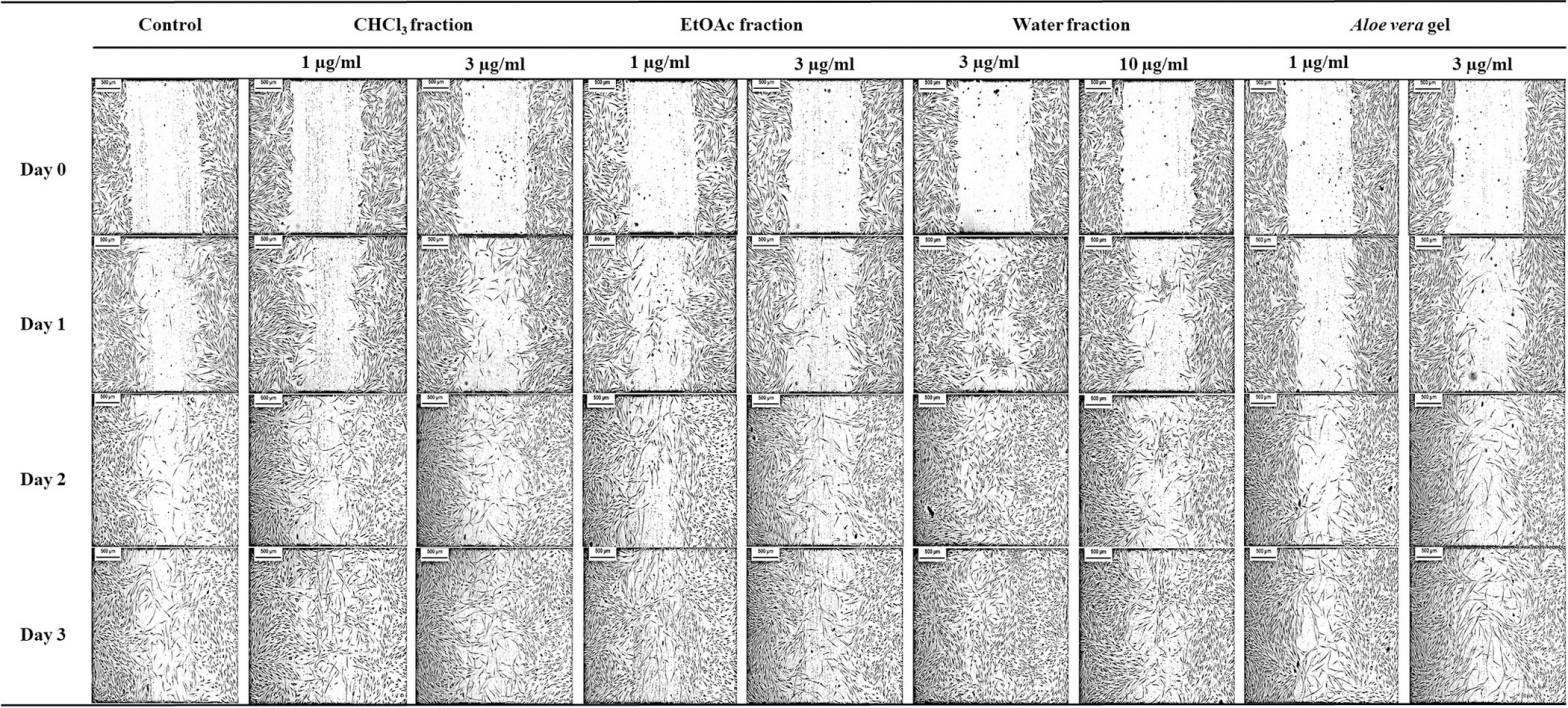
Effect of the CHCl_3_, EtOAc, water fractions and *A*. *vera* gel on HDF migration. Images were captured at day 0 and showed that an artificial wounded monolayer was created using the scratch assay. Then, treatments were applied with 1 and 3 μg/ml CHCl_3_ fraction, 1 and 3 μg/ml EtOAc fraction, 3 and 10 μg/ml water fraction, 1 and 3 μg/ml *A*. *vera* gel and control without treatment. Another set of images of fibroblast cell migration were captured at day 1, 2 and 3 after incubation. The cleared areas represented wound and shaded areas resulting in cell migration, which represents the wound closure.

Cell migration plays a key role in wound healing. In an attempt to investigate the wound healing efficacy of the active fractions and compounds from *D*. *bulbifera*, an artificial wounded monolayer was created using the scratch assay to assess the fibroblast cell migration. Consequently, fractions F1-F3, which resulted in good cell proliferation, were also tested in the cell migration assay. The CHCl_3_ fraction (F1) at 1 and 3 μg/ml and the water fraction (F3) at 3 and 10 μg/ml resulted in the highest cell migration of 100.0% by day 3. However, the EtOAc fraction (F2) (1 and 3 μg/ml) resulted in 100.0% and 83.2% cell migration, respectively, by day 3. Moreover, the water fraction (F3) at 3 μg/ml resulted in 100% wound closure starting on day 2. *A*. *vera* gel (positive control) at 1 and 3 μg/ml resulted in 93.9 and 89.9% lower cell migration, respectively, than F1-F3. The wound closure in the control was 86.3% by day 3 (Table 4 and Fig 2). This result suggested that F1-F3 resulted in higher % cell migration than the positive control and the control. According to the literature, *D*. *villosa* was also found to possess wound healing effect, its leaf extract induced the migration of L929 fibroblast cells as a result of the expression of collagen‑1 and transforming growth factor (TGF)‑beta [30].

**Table 4:** Effect of fractions from *D*. *bulbifera* bulbils on HDF migration.

Sample	Dose (μg/ml)	Length between the scratches (μm)				Rate of cell migration (%)		
		Day 0	Day 1	Day 2	Day 3	Day 1	Day 2	Day 3
Control	-	1428.42 ± 9.67	810.57 ± 7.31	423.25 ± 17.24	195.10 ± 8.65	43.25 ± 0.51^a^	70.37 ± 1.21	86.34 ± 0.61^a^
CHCl_3_ fraction	1	1317.69 ± 10.97	690.45 ± 6.85	278.29 ± 5.46	0.00 ± 0.00	47.60 ± 0.52*^,^^a^	78.88 ± 0.41*^,^^a^	100.00 ± 0.00*^,^^a^
	3	1276.44 ± 2.09	618.37 ± 6.35	345.58 ± 4.58	0.00 ± 0.00	51.55 ± 0.50*^,^^a^	72.93 ± 0.36^a^	100.00 ± 0.00*^,^^a^
EtOAc fraction	1	1307.25 ± 1.38	670.08 ± 10.01	315.79 ± 5.06	0.00 ± 0.00	49.96 ± 0.75*^,^^a^	76.42 ± 0.38*^,^^a^	100.00 ± 0.00*^,^^a^
	3	1339.11 ± 6.36	835.64 ± 16.41	400.41 ± 10.73	224.08 ± 9.76	37.60 ± 1.23*	70.10 ± 0.80	83.27 ± 0.73^a^
Water fraction	3	1358.95 ± 6.85	332.69 ± 28.03	0.00 ± 0.00	0.00 ± 0.00	75.52 ± 2.06*^,^^a^	100.00 ± 0.00*^,^^a^	100.00 ± 0.00*^,^^a^
	10	1311.43 ± 13.33	675.30 ± 21.08	307.59 ± 8.03	0.00 ± 0.00	48.51 ± 1.61*^,^^a^	76.55 ± 0.61*^,^^a^	100.00 ± 0.00*^,^^a^
*A*. *vera* gel	1	1278.53 ± 8.29	872.72 ± 0.91	447.09 ± 10.02	82.38 ± 41.23	35.73 ± 0.07	67.08 ± 0.74	93.93 ± 3.04
	3	1357.91 ± 24.70	825.19 ± 21.12	256.31 ± 0.65	136.23 ± 10.80	39.23 ± 1.56	81.12 ± 0.05	89.97 ± 0.80

*Statistically significant difference between the control and sample, *p* < 0.05 (mean ± S.E.M. of three determinations).

^a^Statistically significant difference between *A*. *vera* gel and the sample, *p* < 0.05 (mean ± S.E.M. of three determinations).

**Fig 3:**
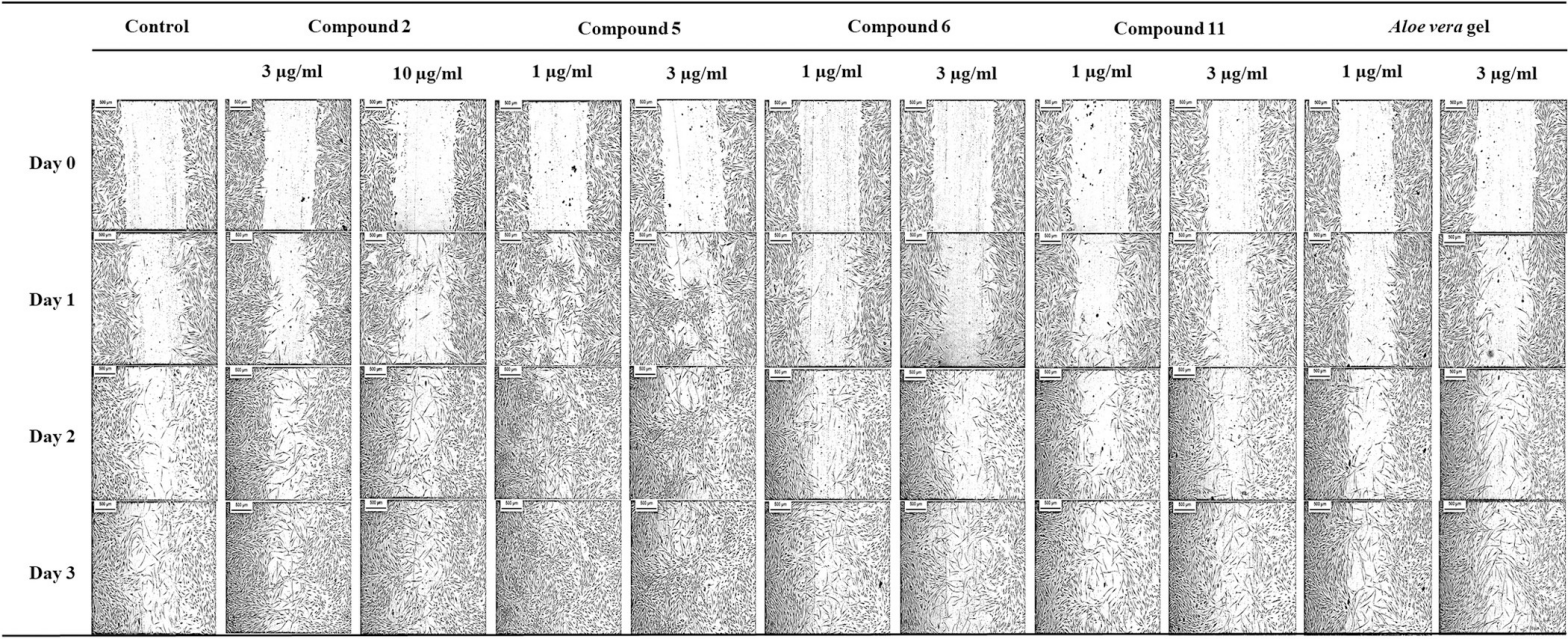
Effect of compounds 2, 5, 6, 11 and *A*. *vera* gel on HDF migration. Images were captured at day 0 and showed that an artificial wounded monolayer was created using the scratch assay. Then treated with 3 and 10 μM 15,16-epoxy-6α-*O*-acetyl-8β-hydroxy-19-nor-clero-13(16),14-diene-17,12,18,2-diolide (**2**), 1 and 3 μM (+)-catechin (**5**), 1 and 3 μM quercetin (**6**), 1 and 3 μM myricetin (**11**), 1 and 3 μg/ml *A*. *vera* gel and control without treatment. Another set of images of fibroblast cell migration were captured at day 1, 2 and 3 after incubation. The cleared areas represented wound and shaded areas resulting in cell migration, which represents the wound closure.

15,16-Epoxy-6α-*O*-acetyl-8β-hydroxy-19-nor-clero-13(16),14-diene-17,12;18,2-diolide (**2**), (+)-catechin (**5**), quercetin (**6**) and myricetin (**11**) also resulted in a good % cell migration. Compound **2** at 3 and 10 μM enhanced migration of HDFs resulted in 100.0% by day 3. Compound **5** (1 and 3 μM) produced 100.0% cell migration by day 2 whereas **6** (1 and 3 μM) produced 100.0% cell migration by day 3. For compound **11,** 1 and 3 μM induced 87.6 and 87.4% cell migration, respectively, by day 3. Therefore, **5** exhibited the highest % cell migration, producing 100.0% wound closure sooner (on day 2) than the other compounds (**2**, **6** and **11**) (Table 5 and Fig 3). In addition, 1 μg/ml compound **5** significantly enhanced fibroblast migration by 2.4-fold compared to that in the control after 24 h. Among the compounds isolated from *D*. *bulbifera*, **5** showed the highest activity in terms of both cell proliferation and cell migration.

**Table 5:** Effects of compounds 2, 5, 6 and 11 on HDF migration.

Sample	Dose (μM)	Length between scratches (μm)				Rate of cell migration (%)		
		Day 0	Day 1	Day 2	Day 3	Day 1	Day 2	Day 3
Control	-	1428.42 ± 9.67	810.57 ± 7.31	423.25 ± 17.24	195.10 ± 8.65	43.25 ± 0.51	70.37 ± 1.21	86.34 ± 0.61
15,16-Epoxy-6α-O-acetyl-8β-hydroxy-19-nor-clero-13(16),14-diene-17,12;18,2-diolide (**2**)	3	1186.61 ± 28.16	19.42 ± 15.81	119.65 ± 21.68	0.00 ± 0.00	47.80 ± 1.33^a^	89.92 ± 1.83*^,^^a^	100.00 ± 0.00*^,^^a^
	10	1344.33 ± 17.82	783.41 ± 7.23	106.51 ± 53.54	0.00 ± 0.00	41.72 ± 0.54^a^	92.08 ± 3.98*^,^^a^	100.00 ± 0.00*^,^^a^
(+)-Catechin (**5**)	1	1361.04 ± 5.81	205.66 ± 3.85	0.00 ± 0.00	0.00 ± 0.00	85.24 ± 0.28*^,^^a^	100.00 ± 0.00*^,^^a^	100.00 ± 0.00*^,^^a^
	3	1393.43 ± 22.98	316.82 ± 20.32	0.00 ± 0.00	0.00 ± 0.00	77.26 ± 1.46*^,^^a^	100.00 ± 0.00*^,^^a^	100.00 ± 0.00*^,^^a^
Quercetin (**6**)	1	1417.45 ± 5.82	815.79 ± 3.77	298.01 ± 16.30	0.00 ± 0.00	42.45 ± 0.27^a^	78.98 ± 1.15*^,^^a^	100.00 ± 0.00*^,^^a^
	3	1414.32 ± 9.96	857.57 ± 11.77	341.37 ± 7.24	0.00 ± 0.00	39.36 ± 0.83*^,^^a^	75.86 ± 0.51*^,^^a^	100.00 ± 0.00*^,^^a^
Myricetin (**11**)	1	1340.15 ± 16.44	815.79 ± 7.31	266.39 ± 3.47	148.67 ± 6.96	32.26 ± 0.61*	77.88 ± 0.29*^,^^a^	87.66 ± 0.58*
	3	1204.36 ± 4.55	758.34 ± 1.81	273.62 ± 8.59	151.15 ± 6.58	37.03 ± 0.15*	77.28 ± 0.71*^,^^a^	87.45 ± 0.55^a^
*A*. *vera* gel (μg/ml)	1	1278.53 ± 8.29	872.72 ± 0.91	447.09 ± 10.02	82.38 ± 41.23	35.73 ± 0.07	67.08 ± 0.74	93.93 ± 3.04
	3	1357.91 ± 24.70	825.19 ± 21.12	256.31 ± 0.65	136.23 ± 10.80	39.23 ± 1.56	81.12 ± 0.05	89.97 ± 0.80

*Statistically significant difference between the control and sample, *p* < 0.05 (mean ± S.E.M. of three determinations).

^a^Statistically significant difference between *A*. *vera* gel and the sample, *p* < 0.05 (mean ± S.E.M. of three determinations).

These findings are in agreement with a previous report in which *D*. *bulbifera* extracts showed wound healing activity in an excision wound model in rats. A high rate of wound contraction and a decrease in the period for epithelialization were observed [14]. (+)-Catechin (**5**), a compound that exhibited the most potent effect on cell proliferation and cell migration, is distributed in a variety of foods and herbs and has been reported to possess antioxidant, anticancer and anti-HIV-1 integrase properties [17,31,32]. Additionally, catechin derivatives have been previously established in an *in vitro* wound healing model. Epicatechin-3-*O*-gallate and 4′-*O*-methylepicatechin-3-*O*-gallate, isolated from the bark of *Parapiptadenia rigida*, exhibited promising wound healing effects in a scratch assay [33]. Regarding quercetin (**6**), our result is consistent with those in the literature and promoted the wound healing process by modulating cells involved in inflammatory. Quercetin-treated rats showed less inflammatory cells, more fibroblast proliferation and more regular collagen deposition [15].

### Antioxidant activity

Free radicals and oxidative reactions are involved in all wound healing processes. Excessive production of reactive oxygen species (ROS) or impaired ROS detoxification results in accelerated inflammation- and oxidative stress-induced cellular damage, which is the main cause of delayed wound healing [34]. Therefore, experimental studies on the elimination of ROS and antioxidants could be an important strategy for healing chronic wounds. To determine the antioxidant activity, the crude extracts, their derived fractions and the isolated compounds **1**–**11** from *D*. *bulbifera* were evaluated to assess their ability to scavenge DPPH and ^•^OH radicals.

#### DPPH radical scavenging assay

DPPH is a synthetic nitrogen-centered free radical that can accept electrons or hydrogen radicals from antioxidants to form a stable molecule. DPPH is widely used to evaluate the scavenging activity of antioxidants because it is simple and highly sensitive [19,35]. As shown in Table 6, all crude extracts and their derived fractions were found to be active against DPPH radicals, with IC_50_ values ranging from 13.20 to 34.14 μg/ml. For the pure compounds, the strongest scavenging effect was observed for compound **11**, with an IC_50_ value of 4.87 μM, which is comparable to that of ascorbic acid (IC_50_ = 3.99 μM), a positive control, followed by compounds **6**, **5**, and **4** (IC_50_ = 5.33, 8.27, and 9.02 μM, respectively).

**Table 6:** Antioxidant activity of extracts, their derived fractions and the compounds isolated from *D*. *bulbifera*, as evaluated by DPPH and hydroxyl radical scavenging.

No	Sample	IC_50_ (μM)	
		DPPH radical	Hydroxyl Radical
1^c^	EtOH extract	34.14 ± 0.68*	79.00 ± 0.78*
2^c^	Water extract	13.20 ± 0.77*	>100
3^c^	CHCl_3_ fraction	13.35 ± 0.37*	>100
4^c^	EtOAc fraction	14.00 ± 0.36*	37.04 ± 0.50*
5^c^	Water fraction	18.83 ± 0.46*	39.31 ± 0.42*
6	8-Epidiosbulbin E acetate (**1**)	80.87 ± 0.48*	>100
7	15,16-Epoxy-6α-*O*-acetyl-8β-hydroxy-19-nor-clero-13(16),14-diene-17,12;18,2-diolide (**2**)	37.57 ± 1.27*	51.90 ± 0.62*
8	Sitosterol-β-D-glucoside (**3**)	>100	>100
9	3,5-Dimethoxyquercetin (**4**)	9.02 ± 0.56*	22.07 ± 0.94*
10	(+)-Catechin (**5**)	8.27 ± 0.44*	19.58 ± 0.27*
11	Quercetin (**6**)	5.33 ± 0.25	16.80 ± 0.33*
12	Kaempferol (**7**)	16.15 ± 0.25*	51.68 ± 0.53*
13	Allantoin (**8**)	>100	>100
14	2,4,3',5'-Tetrahydroxybibenzyl (**9**)	16.20 ± 0.27*	63.84 ± 0.54*
15	2,4,6,7-Tetrahydroxy-9,10 dihydrophenanthrene (**10**)	22.68 ± 0.54*	71.55 ± 1.12*
16	Myricetin (**11**)	4.87 ± 0.41	11.68 ± 0.39*
17	L-Ascorbic acid (positive control)	3.99 ± 0.13	ND
18	BHT (positive control)	ND	4.06 ± 0.41

*Statistically significant difference between the positive control and sample, *p* < 0.05 (mean ± S.E.M. of three determinations).

^c^Concentration of treated samples and IC_50_ expressed in μg/ml.

ND = not determined.

#### Hydroxyl radical (^•^OH) scavenging activity

Hydroxyl radicals are regarded as the most reactive free radicals among ROS and can cause serious damage to biomolecules. They play an important role in inflammation in oxidative stress-induced diseases [36]. The scavenging activity of ^•^OH is commonly used to determine the ability of a substance to scavenge free radicals. Therefore, further confirmation of the abovementioned antioxidant effects of the extracts, their fractions and the compounds isolated from *D*. *bulbifera* were evaluated by determining the ^•^OH scavenging activity. As shown in Table 6, the EtOAc and water fractions possessed inhibitory activity, with IC_50_ values of 37.04 and 39.31 μg/ml, respectively, while the other extracts and fractions showed weak effects.

Among the isolated compounds, **11** showed the most potent inhibitory effect, with an IC_50_ value of 11.68 μM, followed by **6, 5** and **4 (**IC_50_ = 16.80, 19.58 and 22.07 μM, respectively), whereas the other compounds exhibited mild and moderate activities. The results of the ^•^OH assay agree with the radical scavenging results of the DPPH assay: compounds **11**, **6**, **5** and **4**, the flavonoid compounds, possessed potent activity. Altogether, these data suggested that flavonoids are the main active compounds responsible for the antioxidant properties of *D*. *bulbifera*. Clearly, the scavenging effect increases with the number of hydroxyl groups attached to the aromatic B-ring.

## Conclusions

In this study, flavonoid compounds isolated from *D*. *bulbifera* bulbils inhibited NO production and possessed antioxidant properties assessed via DPPH radical and hydroxyl scavenging assays. (+)-Catechin is a major active compound responsible for the wound healing effect of *D*. *bulbifera* and shows high potential for wound healing by promoting the cell proliferation and cell migration of human fibroblasts. This effect may be attributed mainly to its antioxidant properties. These findings provide more evidence to support the traditional use of *D*. *bulbifera* for the treatment of wounds and inflammation.
